# Advanced Biofuels from ABE (Acetone/Butanol/Ethanol) and Vegetable Oils (Castor or Sunflower Oil) for Using in Triple Blends with Diesel: Evaluation on a Diesel Engine

**DOI:** 10.3390/ma15186493

**Published:** 2022-09-19

**Authors:** Laura Aguado-Deblas, Francisco J. López-Tenllado, Diego Luna, Felipa M. Bautista, Antonio A. Romero, Rafael Estevez

**Affiliations:** Department of Organic Chemistry, University of Cordoba, Campus of Rabanales, Ed. Marie Curie, 14014 Córdoba, Spain

**Keywords:** ABE blend, straight vegetable oil, castor oil, sunflower oil, biofuel, diesel engine, electricity generator, exhaust emissions, soot, NO_x_, CO, CO_2_

## Abstract

From a technical and economic point of view, our aim is to provide viable solutions for the replacement of fossil fuels which are currently used in internal combustion diesel engines. In this research, two new biofuels composed of second-generation vegetable oils (SVO),used oil sunflower (SO) or castor oil (CO), and the ABE blend (acetone/butanol/ethanol) were evaluated. ABE is an intermediate product from the fermentation of carbohydrates to obtain bio-butanol. Besides, the ABE blend exhibits suitable properties as biofuel, such asvery low kinematic viscosity, reasonable energy density, low autoignition temperature, and broad flammability limits. Diesel/ABE/SVO triple blends were prepared, characterized and then, tested on a diesel engine, evaluating power output, consumption, and exhaust emissions. The power output was slightly reduced due to the low heating values of ABE blend. Also, engine consumed more fuel with the triple blends than with diesel under low engine loads whereas, at medium and high loads, the fuel consumption was very similar to that of diesel. Regarding exhaust gas emissions, soot wasnotably reduced, and nitrogen oxides (NO_x_) and carbon monoxide (CO_2_) emissions were lower or comparable to that of diesel, while the CO emissions increased. The use of these biofuels allows the replacement of high percentagesof diesel without compromising engine power and achievinga significant reduction in pollution emissions. Furthermore, a notable improvement in cold flow properties of the fuel blends is obtained, in comparison with diesel.

## 1. Introduction

Nowadays, the reduction of greenhouse gas emissions is a “no return” decision in most industrialized countries. Furthermore, the choice of green hydrogen as the main energy vector for the decarbonization of the planet seems definitive. Therefore, it could be assumed that biofuels will play a secondary role in the current research and development priorities for different means of transport, including heavy-duty transportation sector. However, a period of at least several decades is required for the transition from current transport energy sources to hydrogen technology, according to the planning established by the countries involved in this transition process [1]. This long transition period is due not only to the maturity degree of fuel cells and/or electric motor technologies, which can hardly compete with fossil fuels engines, especially in heavy trucks, aviation, or the shipping sector [2], but also to the enormous car fleet operating across the world [3]. Thus, a good solution for fossil fuel substitution is keeping biofuels active during this transition period [4].

Nevertheless, the replacement of fossil fuels by other renewable fuels exhibits many different difficulties. To fulfill the energy purposes, important amounts of agricultural resources are necessary [5], since the liquid biofuels considered for these processes are mainly bioethanol and several vegetable oils. In this sense, there is an urgent priority to promote the optimization of agricultural crops that can be destinated to the production of liquid biofuels, with the use of inedible and higher-yielding seeds, or making use of wastes [6]. On the other hand, the gasoline car engine vehicle fleet does not present special difficulties due to using a high percentage of fossil fuel substitution via bioethanol [7].

Straight vegetable oils (SVOs) cannot be directly used in the compression-ignition (CI) diesel engines since they exhibit higher kinematic viscosity values than fossil diesel [8]. For this reason, despite SVOs havevery similar physico-chemical properties to those of fossil diesel, they require previous treatment to reduce their viscosity values and fulfill the European Regulation EN 590. A very simple solution commonly employed is the alkaline transesterification of the triglycerides that constitute vegetable oils or animal fats, with methanol. This reaction givesrise to a mixture of methyl esters of the fatty acids (FAME), usually known as biodiesel [9]. However, the formation of glycerol as by-product in the biodiesel production is its main limitation. The glycerol cannot be used in the engines. In fact, the amount of glycerol in biodiesel must be lower than 0.25 wt.%, according to EN 14214 [10,11]. Thus, the enormous amounts of glycerol generated cannot be absorbed by the current market, which has caused the infeasibility of the biodiesel production process and so, a long delay in the process for substituting fossil fuels. Consequently, the search for new biofuels based on vegetable oil, is still an urgent priority [10,11,12]. 

In the last two decades, a great effort has been made in order to find alternatives to reduce the viscosity of triglycerides, allowing the use of SVOs as biofuels [11]. Among the different options studied, such as the obtention of biodiesel-like biofuels that integrate glycerol as a soluble derivative [12], and green diesel production [13,14,15], the use of biofuels obtained by blending an SVO with organic solvents (having lower viscosity and cetane number) (LVLC) has gained great attention [16]. This methodology does not entail chemical treatments of vegetable oils, and the costs are limited to the kind of oil and organic solvent employed. In addition, no residues are generated. Thus, it has been reported that the use of pine oil as an LVLC solvent allows the use of very high viscosity oils, such as castor oil, as a biofuel in CI engines [17]. In this way, the high viscosity of neat castor oil, 226.2 cSt, is balanced with the low viscosity of pine oil, 1.3 cSt. However, bothlow cetane number and calorific value of pine oil limit its ratio by to up 30% (by volume) [18]. Analogously, various organic compounds with low molecular weight and renewable character are currently being applied as LVLC solvents [19,20]. In any case, the final adequate proportion of the LVLC solvent is determined not only by its influence on the viscosity of the double SVO/LVLC mixture but also by its cetane number, which can negatively affect engine operation. The use of Diesel/SVO/LVLC triple blends would allow to reduce the amount of fossil fuel employed. Besides, the use of these triple blends has been found to notably reduce polluting emissions. Thus, several bio-alcohols, such as n-propanol, n-butanol, and n-pentanol, have been studied for this purpose [21,22,23].

Likewise, other shorter chain alcohols, such as ethanol or isopropanol, have also been evaluated in ternary blends with SVO and diesel fuel. However, the low calorific power of ethanol and 2-propanol (27 and 33 MJ/kg, respectively) constitutes the greatest limitation for its use in triple blends with oils [24]. However, other chemicals obtained from bioethanol can be employed as LVLC solvents. Among these compounds, diethyl ether (DEE) has been the most studied. Thus, it has been described as a fuel in CI engines inblends withcashew nut shell oil [25,26], neat cottonseed oil [27], bael oil [28], sunflower oil, and castor oil [29]. In spite of having some favorable properties for blending, such as a very low kinematic viscosity, low autoignition temperature and high oxygen content, its low calorific power limits the percentage of substitution with fossil fuel via the DEE/oil blend. However, the incorporation of DEE in a triple blend with a SVO (diesel/vegetable oil/DEE) allows for a substitution with fossil diesel of up to 40% by volume, not only promoting good results in terms of engine power and emission reductions, but also leading to an improvement in the cold flow behavior of fuel [30]. Many other solvents have also been employed as an LVLC in triple blends with diesel and oil, such as ethyl acetate (EA) [31,32], diethyl carbonate [33], dimethyl carbonate [34], and acetone [35]. In general, a fossil diesel substitution above 40% was obtained, attaining a reduction in pollutant emissions without diminishing the power generated by the engine.

Bio-butanol has been demonstrated to be a potential alternative biofuel in internal combustion engines [36], being successfully applied in triple blends with diesel and SVO [21,22,23,37,38,39,40,41,42,43,44]. It can be obtained from renewable resources, such as the cellulose contained in a wide variety of waste feedstocks, through a typical acetone-butanol-ethanol (ABE) fermentation process, where the typical ratio of ABE is 3:6:1 [45]. However, bio-butanol has not been produced at a large scale due to the high economic and energy costs for recovering bio-butanol from ABE. Also, butanol obtained from ABE fermentation has low productivity (12–18 g/L) [45,46]. Thus, the direct use of ABE as LVLC instead of butanol would increase the economic possibilities for its application as a biofuel [47,48]. In fact, the use of ABE in blends with both gasoline and diesel has already been reported [46]. The studies indicate that the addition of ABE leads to fewer exhaust emissions (CO, NO_x_, and soot) as well as an improvement in engine performance.

To the best of our knowledge, there are no previous studies on the use of ABE as a solvent of vegetable oils for use in diesel engines. Therefore, this paper aims to evaluate ABE as a renewable solvent of second-generation vegetable oils to produce new biofuels that can to reduce (as much as possible) the amount of diesel employed, keeping good engine performance.

Herein, sunflower oil and castor oil were chosen as second-generation vegetable oils since these are easily available and are not competing with food usage. Castor oil is the only inedible vegetable oil available on an industrial scale (about 220,000 tons/year) [49], so it could be applied massively and immediately as a biofuel. Sunflower oil is here used as a reference for waste cooking oil in order to avoid reproducibility problems due to the different origins of waste oils.

Firstly, the suitable proportions of each component in the double blends were determined, and then, the most relevant fuel properties of the different diesel/ABE/SVO triple blends were studied. Secondly, some of the most important parameters for engine performance, i.e., fuel consumption and power output, as well as exhaust emissions from engine diesel fueled with these diesel/ABE/SVO triple blends, were evaluated.

## 2. Materials and Methods

The different proportions of each component in the ABE/SVO double blends were chosen based on kinematic viscosity. In order to evaluate their influence on the operation of a diesel engine, some of the most relevant fuel properties, including viscosity, density, calorific value, cetane number, pour point, and cloud point, were determined. Then, power output, fuel consumption, and polluting emissions (soot, CO, NO_x,_ and CO_2_) were analyzed.

### 2.1. Preparation of ABE/Vegetable Oil Double Blend and Diesel/ABE/Vegetable Oil Triple Blends

Sunflower oil (food grade) was obtained from a local market; castor oil, butanol (B), and ethanol (E) (≥99.5% purity) from Panreac, Castellar Del Valles, Spain. Acetone (A) was acquired from Sigma-Aldrich Chemical Company (St. Louis, MO, USA). Diesel was obtained from Repsol service station.

**Figure 1:**
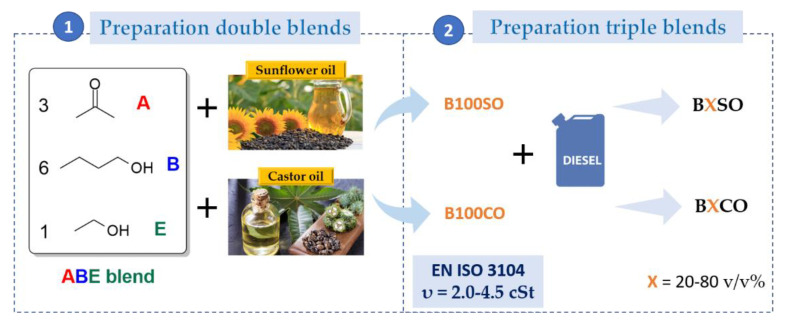
Procedure employed for the preparation of (bio)fuel blends.

A scheme of experimental procedures for fuel blend preparation is shown in Figure 1. Firstly, double blends were prepared according to the viscosity requirements of the European petro-diesel standard, EN-590 ISO-3104 (υ = 2.0–4.5 cSt). Thus, a standard ABE blend, typically in the volumetric ratio of acetone/butanol/ethanol 3:6:1, was mixed with either sunflower or castor oil in different proportions of 10, 30, 50, 60, and 70 *v*/*v*%. The double blends containing sunflower oil and castor oil were termed B100SO and B100CO, respectively. Secondly, the triple blends were prepared by adding the double blends selected to fossil diesel in proportions from 20 to 80 *v*/*v*%. The triple blends were denoted as BXSO or BXCO (X = 20, 40, 60, and 80), where X is the percentage of biofuel (ABE/SVO blend) added to the diesel. For comparison, pure diesel (B0) was also studied as a reference.

### 2.2. Physico-Chemical Characterization of the Biofuel Blends

Kinematic viscosity was determined following the specifications established by the European standard (EN 590 ISO 3104), using an Ostwald-Cannon-Fenske capillary viscometer (Proton Routine Viscometer 33200, size 150), working at 40 °C. In this respect, it was determined: the flow time (t), expressed in seconds, required for a certain volume of liquid to pass under gravity between two marked points on the instrument, placed in an upright position. The kinematic viscosity (ʋ), expressed in centistokes (cSt), was obtained from Equation (1):

ʋ = C·t,(1)

In this equation, C is the calibration constant of the measurement system, specified by the manufacturer (0.037150 (mm^2^/s)/s 40 °C), following the methodology described in previous works [29,34]. The viscosity values reported here are the average of the three determinations, following the standard ASTM (American Society for Testing and Materials) D2270-79 method for calculating the viscosity index from kinematic viscosity at 40 °C.

Density values were determined at 15 °C following the EN ISO 3675 test method.

Cold flow properties are responsible for the solidification of the fuel under cold operating conditions. At low temperatures, fuel crystallization results in clogging the fuel lines and filters, which hinders the engine from starting due to the lack of fuel. The cold flow properties were determined, as specified by standard methods, EN 23015/ASTM D2500, for cloud point, and ISO 3016/ASTM D97, for pour point. A previously reported methodology was employed [29,32].

Every value is obtained as average of duplicate determinations.

The calorific value (CV), or heat of combustion, expressed in MJ/kg, was determined theoretically, according to the volumetric concentration of each component in the blend from the following Kay Mixing rule (Equation (2)):

(2) $$\mathrm{CV}=\sum_{i}CViXi$$

where *CVi* is the calorific value of each component, and *Xi* is the volumetric fraction of every component.

Analogously, the Cetane Number (CN) was estimated using Equation (3):

(3) $$\mathrm{CN}=\sum_{i}CNiXi$$

where *CNi* is the cetane number of each component, and *Xi* is the volumetric fraction of every component [50]. 

### 2.3. Performance and Exhaust Emissions of a Diesel Engine Electric Generator Fueled with the Different ABE/SVO Double and Diesel/ABE/SVO Triple Blends

Energy performance and exhaust emissions were analyzed following the previously described experimental methodology [29,32,33,34] on a 4-stroke and single-cylinder engine (dimensions: 78 mm bore and 67 mm stroke), with a forced air-cooling system by flywheel fan. The main specifications of the engine are shown in Table 1. Besides the biofuel blends, pure biofuels and conventional diesel were also studied for comparison.

This engine operates at a crankshaft constant rotation rate under different load conditions (0, 1, 2, 3, 4 m and 5 kW), i.e., different degrees of demanded electrical power to the engine provided by connecting the heating plates (of 1000 watts each). Each test was performed by employing a volume of 0.5 L. Before each test, the engine was made to run for 20 min to reach stable conditions. Also, the system was purged by fueling the engine with diesel and working it for 20 min.

**Figure 2:**
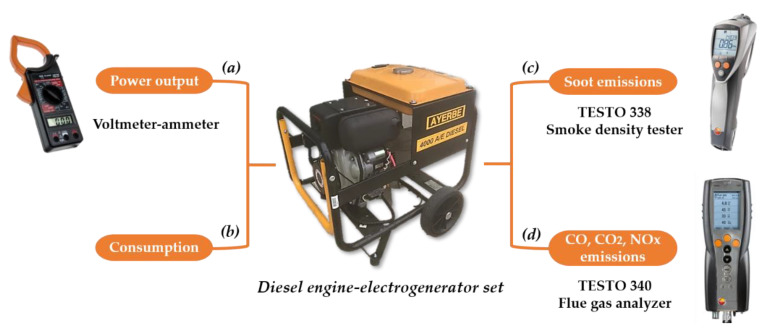
Experimental methodology employed for the evaluation of the biofuels using a Diesel Engine-Electrogenerator Set based on (**a**) power output from a voltmeter–ammeter devise, (**b**) fuel consumption, (**c**) soot emissions using a smoke density tester, and (**d**) CO, CO_2_, and NO_x_ emissions, using a flue gas analyzer.

The electrical power generated by the engine was obtained from the product of the potential difference (or voltage) and the electric current intensity (or amperage), Equation (4), both measured by using a voltmeter-ammeter (Figure 2a):

Electrical Power Generated (Watts) = voltage (Volts) × amperage (Amps)(4)

Fuel consumption was measured by determining the time taken by the diesel generator to consume certain amounts of (bio)fuel (0.5 L). The fuel consumption was expressed as the brake-specific energy consumption (BSFC) in g/h·kW, which is the mass of fuel consumed per hour and per kW of power generated by the engine. The BSFC measurements were carried out at engine loads of 1, 3, and 5 kW, which represent low-, medium-, and high-power demands. Experimental tests were done in triplicate, and the results are shown as an average of the three measurements. The errors are calculated as standard deviation and represented as error bars.

The degree of pollution was determined based on the opacity of the smoke and the carbon monoxide (CO), nitrogen oxides (NO_x_), and carbon dioxide (CO_2_) emitted by the engine during the combustion process. For the opacity measurements, an opacimeter-type TESTO 338 smoke density tester was used, according to the standard method ASTM D-2156 (Standard Test Method for Smoke Density in Flue Gases from Burning Distillate Fuels) (Figure 2c). The instrument calculates the smoke density from the level of soot on a filter paper. The smoke emissions are expressed as a Bosch number, which is a standardized unit with a measurement range from 0 to 2.5, with 0 being absolute clarity (on the paper) and 2.5 as 100% blackening. The CO, NO_x_, and CO_2_ levels in the exhaust gas were measured with a Testo 340 flue gas analyzer (Figure 2d). The detected amounts of CO and NOx are expressed in ppm (parts per million), while CO_2_ is expressed as volumetric percentage. The analyzers were calibrated with zero gas before each test. Table 2 shows the accuracy of the measurements of the different parameters.

## 3. Results and Discussion

### 3.1. Physico-Chemical Properties of ABE/SVO Double Blends, and D/ABE/SVO Triple Blends

Table 3 collects the most significant physico-chemical properties of the reactants employed in the blends, i.e., fossil diesel, sunflower oil, castor oil, acetone, butanol, and ethanol.

Regarding the viscosity values of the blends (Table 4), a decrease in the viscosity was observed as the amount of ABE was added to the SVO (independently of the SVO employed). However, the influence of the ABE on reducing the viscosity of the castor oil was stronger than on the sunflower oil since adding a 30% ABE blend to CO promoted a decrease in the viscosity values of around 85% (from 226.2 to 32.44 cSt), whereas the same amount of ABE reduced the viscosity of SO by around 77% (from 37.8 to 8.6 cSt). The viscosity values required by UNE EN 14214 ISO 3104 were achieved with proportions of ABE/SO 50/50 (υ = 4.30 cSt) and ABE/CO 70/30 (υ = 3.42 cSt). It is essential to control the viscosity values of the blends since viscosity constitutes a fundamental parameter on the quality of fuel atomization and the combustion process. Thus, the maximum and minimum limits for the viscosity of a fuel are required to ensure that the engine works without any risk.

Once the optimal percentage of each biofuel (ABE/SVO blends) was selected, triple blends were prepared by adding the different biofuels to fossil diesel (D) in proportions from 20–80% by volume. The kinematic viscosity values of these triple blends, as well as several physico-chemical properties, such as the density, the cloud point, the pour point, the calorific value, and the cetane number, are shown in Table 5 (blends with SO) and Table 6 (blends with CO).

On one hand, the addition of the biofuel blend (ABE/SVO) to the pure diesel fuel generated an increase in both density and viscosity values due to the higher viscosity of the B100 blends in comparison to that of the fossil diesel (B0, 3.20 cSt). Nevertheless, these viscosity values comply with the European regulations EN 590, which establish that viscosity at 40 °C must fall within the range of 2.0–4.5 cSt.

On the other hand, the addition of B100, either with SO or CO, improves the cold flow properties of diesel. In the blends with sunflower oil (Table 5), the cloud point ranges from −6 °C (diesel) to −10.6 °C (B80). Analogously, the pour point goes from −16 °C (fossil diesel) to −23.5 °C (B80). B100 exhibited the best cold flow properties, with values of −13 and −24 °C for cloud point and pour point, respectively. In the case of the castor oil blends, the results obtained are even better since the B80 blend exhibited temperatures for the cloud point and pour point of −16 °C and −28 °C, respectively (Table 6). Regarding the calorific value and cetane number of the triple blends, these values decrease as the percentage of biofuel in the triple blends increases. This fact is expected considering the lower energetic density and cetane numbers of the biofuel components.

### 3.2. Mechanical Performance of a Diesel Engine Fueled with the Different (Bio)Fuel Blends

**Figure 3:**
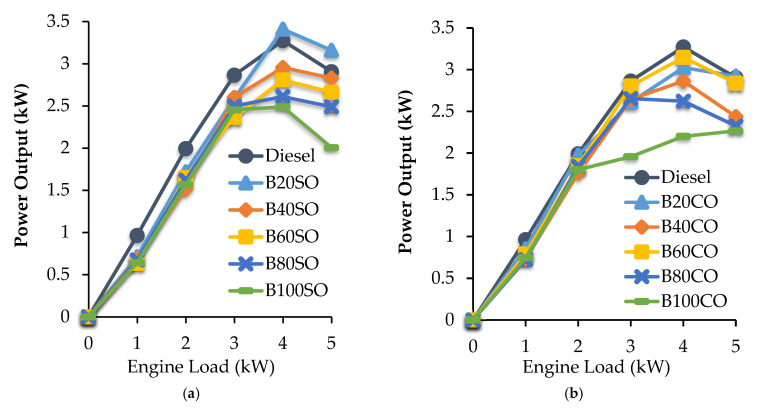
Power generated by the engine at different engine loads using the triple blends: diesel/ABE/sunflower oil (**a**) and diesel/ABE/castor oil (**b**).

For evaluating the maximum amount of biofuel that can be incorporated into diesel without losing mechanical efficiency, it is important to determine the power output of the engine fueled with the different (bio)fuels. Figure 3 displays the power output at different engine loads (from 0 kW to 5 kW) for the triple blends containing either sunflower oil (Figure 3a) or castor oil (Figure 3b). For comparative purposes, diesel fuel and the pure biofuels (B100SO or B100CO) are also included.

In general, as the engine load increased from 0 kW to 4 kW, higher power output values were obtained, while power output was stabilized from 4 to 5 kW. Furthermore, the power output slightly decreased as the percentage of ABE/SVO biofuel added to diesel increased, which can be mainly attributed to the lower energy content of the studied blends in comparison to commercial diesel (Table 5 and Table 6). This behavior is in agreement with previous studies related to the use of ABE blends [51,52,53]. However, the influence of any other operational parameters on engine performance cannot be ruled out. Even so, the power losses obtained for those blends containing small proportions of biofuel are not significant. In fact, the use of the B20 blends gave rise to very similar behavior to that obtained with diesel, especially at high engine loads (4 and 5 kW). Regarding the kind of oil employed, big differences were not observed, probably due to their similar physico-chemical properties.

### 3.3. Brake-Specific Fuel Consumption (BSFC)

Consumption is a very important parameter to evaluate the viability of a (bio)fuel intended to replace fossil diesel in the current fleet of vehicles. Thus, the lower the BSFC for a given power output, the more efficient the engine will be.

**Figure 4:**
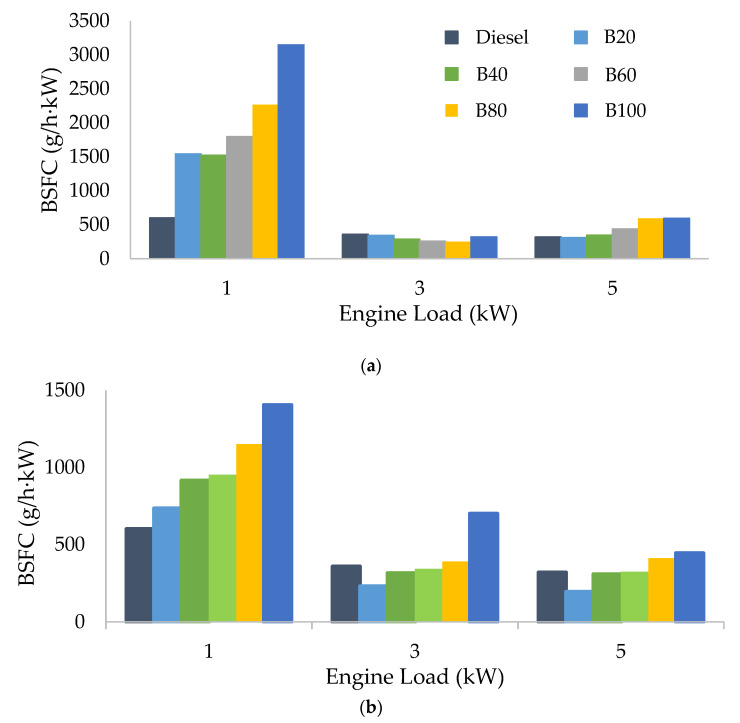
Effect of (**a**) ABE/SO and (**b**) ABE/CO biofuels on BSFC (g/h·kW) at low, medium, and high engine loads (1, 3, 5 kW).

Figure 4 shows the variation of BSFC at low (1 kW), medium (3 kW), and high (5 kW) engine loads for D/ABE/SO blends (Figure 4a) and D/ABE/CO blends (Figure 4b).

As can be seen in Figure 3a,b, the BSFC values decrease as the engine load increases from 1 kW to 3 kW and, afterwards, remains practically constant. The drop in the BSFC values as the engine load increases is associated with a higher temperature inside the cylinder that enhances the combustion process [54]. Overall, the results show that an increment in biofuel content in the blends, from 20% (B20) to 100% (B100), entails a higher BSFC for all triple blends, whether composed of SO or CO. This fact is expected based on the lower calorific values of acetone, butanol, and ethanol in comparison with diesel (Table 3). Indeed, triple blends exhibit 3–12% (SO blends) and 4–15% (CO blends) less energy content than diesel.

Additionally, as ABE content in the blends increased from B20 to B100, other factors, such as density and viscosity, get higher and higher, leading to an increase in BSFC since more fuel is required by the engine to produce the same power. This behavior is mainly observed at low engine loads (1 kW), while the BSFC values for the blends at medium (3 kW) and high engine loads (5 kW) were very similar to that of diesel. Comparing the vegetable oils employed, the blends composed of sunflower oil (D/ABE/SO) yielded slightly lower BSFC values than their counterparts with castor oil (Figure 3a,b). This would be a consequence of the higher energy density of the fuels composed of sunflower oil, which improves the power output of the engine. Also, the lower cetane number of the castor oil blends prolongs the ignition delay, contributing to a higher amount of fuel burned in the premixed combustion phase [34]. In this sense, the results obtained here are consistent with those reported in recent studies [51,55].

### 3.4. Exhaust Emissions from Diesel Engine

#### 3.4.1. Soot Emissions

**Figure 5:**
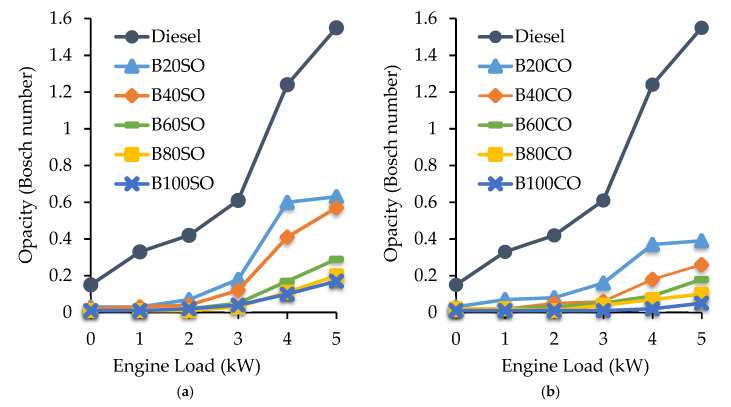
Soot emissions (Bosch number) generated at different engine operating powers (from 0 to 5 kW) with the triple blends: diesel/ABE/sunflower oil (**a**) and diesel/ABE/castor oil (**b**).

The soot values, as a function of engine load, obtained with the different (bio)fuel blends containing SO (Figure 5a) or CO (Figure 5b), are plotted in Figure 5. The results show that all D/ABE/SVO blends promote a significant reduction in smoke emissions as compared to those obtained with conventional diesel. This reduction becomes higher and higher as the volume of the renewable compounds (ABE and vegetable oils) in the triple blend increases. The more pronounced decrease in soot takes place with only a 20% biofuel incorporation into diesel, i.e., the B20SO and B20CO blends emit up to 63 and 75% less soot than diesel, respectively. Lee et al. observed similar behavior with the addition of 10 and 20% ABE to diesel [53]. This is explained by the high oxygen content in the biofuels, which promotes the oxidation of C, resulting in a better combustion process [29,32,33,34,35]. Moreover, the low cetane number in the blends, as well as the higher volatility of ABE in comparison to diesel, promote a higher proportion of fuel burning in the premixed combustion phase, which increases the oxidation of the soot particles [53]. When both vegetable oils are compared, it can be observed that the blends containing castor oil generated less soot than those containing sunflower oil. Based on the above-mentioned results, this fact can be explained by the following reasons: (1) the castor oil molecule exhibits an additional hydroxy group; (2) CO blends contain more ABE within their composition; (3) the cetane number of the CO blends is lower than that of SO; and (4) the lower amount of unsaturation in the ricinoleic acid of the castor oil compared to the linoleic acid in the sunflower oil since the decomposition of unsaturated compounds gives rise to polycyclic aromatic hydrocarbons (PAHs), which are subsequently transformed into soot particles [56].

#### 3.4.2. Carbon Monoxide (CO) Emissions

**Figure 6:**
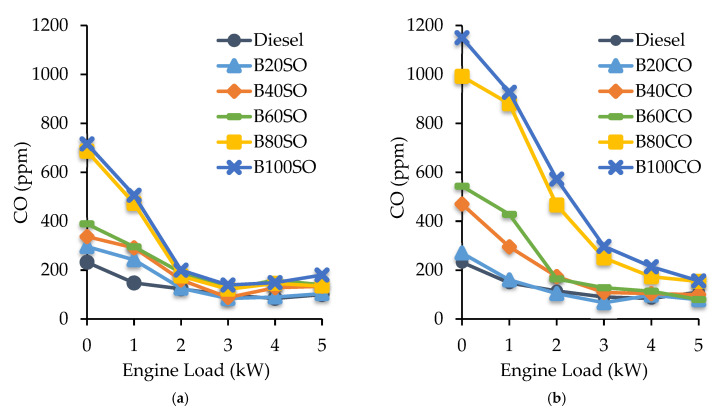
Carbon monoxide (CO) emissions (ppm) generated under different engine operating powers (from 0 to 5 kW), using the triple blends: diesel/ABE/sunflower oil (**a**) and diesel/ABE/castor oil (**b**).

The CO amounts (in ppm) detected in the exhaust emissions under different engine loads fueled with triple blends are shown in Figure 6a (D/ABE/SO) and Figure 6b (D/ABE/CO). Generally, the CO emitted by all of the triple blends was greater than that of diesel, increasing as the percentage of biofuel in the blend increased. This can be explained by the fact that, although a high oxygen content in the fuel offers a high combustion efficiency, the presence of oxygen also reduces the gross heating value of the fuel, which decreases the combustion temperature and retards the oxidation reaction. Additionally, other factors, such as a lower cetane number and higher latent heat of vaporization, as a result of the incorporation of ABE into the blends, also lead to a reduction in combustion temperatures [57]. In fact, for the castor oil blends, which exhibited lower calorific and cetane number values, as well as higher amounts of ABE than their counterparts with SO, showed higher CO emissions. Hence, the B80 and B100 blends generated the highest levels of carbon monoxide, with the B100 blend containing castor oil outperforming diesel in CO emissions to a higher extent, i.e., between 48.7% and 79.8% higher. Consequently, as the amount of biofuel decreases in the blend (B20–B60), the CO emissions become similar to that obtained with diesel. As with the aforementioned, this behavior is due to these blends exhibiting physico-chemical properties closer to those of diesel.

In addition, CO emissions decrease as the engine load of the engine increases. Indeed, the CO emission values for the triple blends were very similar to those obtained for diesel at engine loads of 2 kW upwards, except for the B80CO and B100CO blends, which emit more CO than diesel, reaching similar values only at 5 kW. The higher CO emissions revealed at lower loads are related to higher fuel consumption, which results in a richer air–fuel mixture.

#### 3.4.3. Carbon Dioxide (CO_2_) Emissions

**Figure 7:**
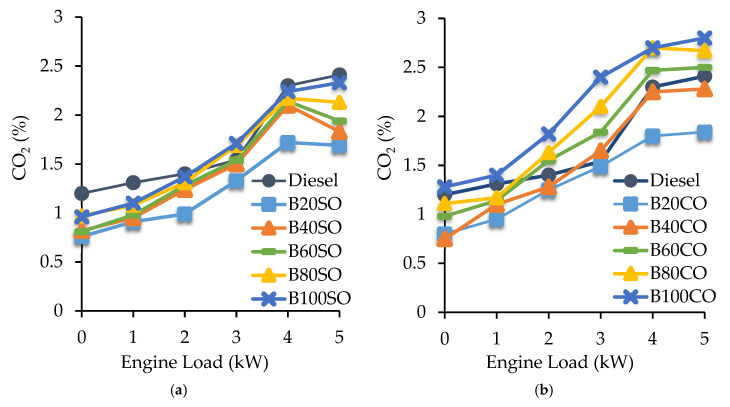
Carbon dioxide (CO_2_) emissions (%) generated under different engine operating powers (from 0 to 5 kW) using the triple blends: diesel/ABE/sunflower oil (**a**) and diesel/ABE/castor oil (**b**).

CO_2_ emissions constitute an important parameter for the evaluation of fuels since they currently are responsible for around 80% of the total greenhouse gas (GHG) emissions. The CO_2_ values obtained from the D/ABE/SVO triple blends under different power demands of the engine are depicted in Figure 7. The results obtained show that, independently of the fuel employed, either fossil diesel or the triple blends, the CO_2_ emissions increase as the engine load increases. However, very good results in terms of CO_2_ emissions were obtained with D/ABE/SO (Figure 7a) since, regardless of the blend (from B20 to B100) and the engine load employed, the CO_2_ emissions were lower than those obtained with diesel. Regarding the D/ABE/CO blends, only B20 improved on the results obtained with diesel. This can be explained because of the higher percentage of ABE incorporated into the castor oil blends (70%), which results in a higher content of oxygen in comparison to the sunflower oil blends (50% ABE). Likewise, an inverse correlation between CO_2_ and CO emissions (Figure 5 and Figure 6) was observed. As the engine load increases, the temperature inside the cylinder also increases, improving the combustion process and promoting the transformation of CO to CO_2_.

#### 3.4.4. Nitrogen Oxides (NO_x_) Emissions

**Figure 8:**
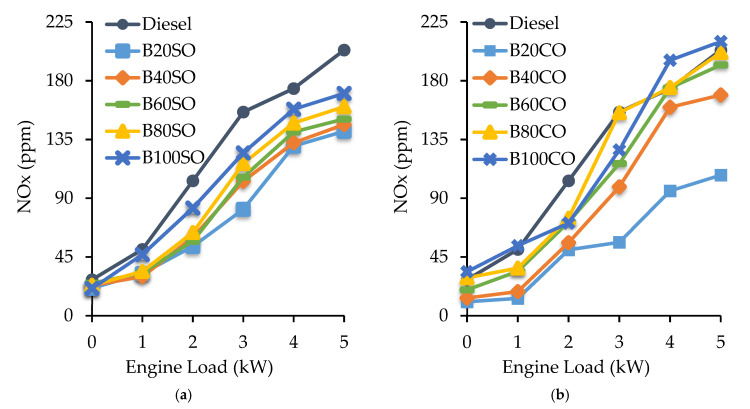
Nitrogen oxides (NO_x_) emissions (in ppm) generated under different engine operating powers (from 0 to 5 kW), depending on the triple blends: (**a**) Diesel/ABE/sunflower oil and (**b**) Diesel/ABE/castor oil.

The NO_x_ amounts emitted by the engine under different engine loads for all the blends studied are shown in Figure 8. A general trend is observed where NO_x_ emissions increase with both the engine load and the biofuel concentration, i.e., the highest NO_x_ emissions were observed for the B100 blends at an engine load of 5 kW. Nevertheless, most of the blends studied emitted less NO_x_ than diesel, which has been associated with the charge cooling effect that reduces the peak temperature and, consequently, the NO_x_ emissions [53]. The increase in NO_x_ emissions as the proportion of biofuel increases is generally attributed to the higher oxygen content in the blends. Also, the increment in the engine load from 0 to 5 kW increases the in-cylinder temperature, favoring the formation of NO_x_ [58]. In the case of blends with SO, the emissions are always lower than those obtained with fossil diesel. Regarding the blends with CO, the B80 and B100 blends exhibited NO_x_ emission values higher than those exhibited by fossil diesel, especially under high engine loads (Figure 7b). However, the B20CO blend showed the lowest NOx emissions among all the blends investigated, emitting 47.1% and 70.3% less NOx than diesel under minimum (0 kW) and maximum engine power (5 kW), respectively. In the mixtures containing sunflower oil, good results are also obtained, with a reduction in the emissions down to 24.3% at 0 kW and 47.8% at 5 kW [46].

#### 3.4.5. Comparison with Previous Results

Our research group reported the effects of adding acetone (ACE) to sunflower or castor oil when using these in blends with diesel in a diesel engine [35]. These results reveal that the blends containing up to 16–18% ACE and 22–24% SVO exhibit an excellent engine performance, producing similar engine power to diesel, with slightly higher fuel consumption and considerable reductions in soot emissions, as well as excellent cold flow properties were also obtained from these triple blends. However, the low calorific value and cetane number, as well as the high volatility of acetone, limit the percentage employed due to engine knocking problems. Ethanol, which is a biofuel that is widely used, exhibits low solubility in diesel and so it can be blended with diesel by up to 20%. However, the percentage of incorporation of ethanol can be increased by blending it with castor oil [59]. For its part, butanol can be blended with diesel at any concentration and exhibits favorable physical and chemical properties for its use as a biofuel [60,61,62]. In fact, it has been reported in a great number of publications that the use of butanol-diesel blends significantly reduces soot emissions, with CO, hydrocarbon (HCs), and NO_x_ emissions also slightly lower than diesel [61,62,63]. Therefore, it is interesting to highlight the potential of ABE blends as an alternative biofuel due to its high butanol content. Indeed, ABE as a biofuel has attracted the attention of many researchers [46,48,64].

However, there are only a few works related to the study of diesel engine performance fueled with diesel/ABE blends and the analysis of their polluting emissions (Table 7). Lee et al. [53] tested the effect of adding 10 and 20% ABE to diesel, and they found higher NO_x_ emissions and significantly lower soot emissions for those fuels containing ABE in comparison to diesel. In this study, it was also concluded that NO_x_ emissions can be reduced by tuning the injection timing. Also, they observed a slight reduction in power output due to lower energy density. Similarly, Lin et al. [65] reported a reduction in soot formation due to the shorter burning duration and stronger premixed burning of the ABE blends. Also, the NO_x_ emissions increased by up to 20 *v*/*v*% with ABE addition. Algayyim et al. found that the addition of 10% and 20% ABE to diesel blends leads to a 20% reduction in unburned hydrocarbons and lower CO emissions in comparison with those of diesel, while NO_x_ emissions slightly increased [51,52]. Chang et al. carried out a study on the effect of a water-containing ABE (in a ratio 5:14:1) as an additive to a biodiesel-diesel blend in a diesel engine [55]. The blends containing 25% of the water-containing ABE solution significantly decreased NO_x_ emissions by 4.30–30.7%, PM emissions by 10.9–63.1%, and PAH (polycyclic aromatic hydrocarbon) emissions by 26.7–67.6% compared to the biodiesel–diesel blends and regular diesel, respectively. In addition, the addition of the water-containing ABE solution caused a higher BSFC and better brake thermal efficiency (BTE).

Here, the incorporation of ABE into the diesel/SVO blends allows lower NOx emissions than with diesel, being lower as the proportion of ABE grows higher. It is remarkable that small percentages of these new biofuels are able to notably reduce soot emissions. In fact, the B40SO blend, which contains the same amount of ABE (20%) than some blends reported in the literature, reduces soot and NOx emissions by 69% and 30%, respectively, with respect to diesel (Table 7). Moreover, CO_2_ emissions are 28% lower than diesel with this blend, in exchange of slight losses to power output. In this case, not reported until now, the incorporation of vegetable oils to the ABE blend presents a series of advantages since biofuels exhibit improved fuel properties, with a higher diesel replacement achieved without a significant impact on engine performance.

## 4. Conclusions

In this study, the viability of using an ABE mixture (acetone/butanol/ethanol) as a solvent for vegetable oils in triple blends (with diesel) was investigated. The most relevant physico-chemical properties of these triple blends were determined. In addition, the influence of these new biofuels has been evaluated using a diesel engine by studying the resulting power output, fuel consumption, and exhaust emissions (unburned hydrocarbons, CO, CO_2_, and NO_x_).

The ABE solvent successfully reduces the viscosity values of the vegetable oils down to the limits required by the European Standard EN 590. Moreover, a notable enhancement of the cold flow properties was attained, especially for the blends with a high amount of biofuel. Therefore, engines fueled with these blends can run more effectively in cold climates than those fueled by diesel and biodiesel. Despite reductions in the calorific value and cetane number, as well as an increment in the density and viscosity values for the triple blends, which occurs as the amount of biofuel in the blends increases, the results related to power output and fuel consumption obtained for the triple blends were similar to or even better than those obtained with diesel, mainly under high engine loads, regardless of the vegetable oil used.

The great potential of biofuels to reduce soot emissions in diesel engines has been demonstrated. This is mainly because of the high oxygen content and lower cetane number in the blends, which promotes an improvement in the combustion reaction. Moreover, the NOx and CO_2_ emissions were comparable or even lower than diesel ones, except for the blends B60CO, B80CO, and B100CO, which emitted a higher amount of CO_2_ than diesel. On the other hand, high emission levels of CO at low engine loads were obtained, mainly for those blends composed of a high proportion of biofuel, i.e., 40% or more (B40-B100 blends). This fact, along with the high consumption obtained, seems to indicate a poor combustion process for the blends at lower engine loads. However, as the engine load increases, an improvement in the combustion process was obtained, probably due to the high temperature reached in the cylinder, among other factors.

The results obtained here confirm the efficacy of these new biofuels in advance of the necessary energy transition since the D/ABE/SVO blends can be successfully employed in current engines without modifications. Moreover, fuel blending is a simple but promising methodology for obtaining new biofuels at a lower cost than other biofuels obtained by chemical treatments, with them also being totally compatible with the environment. The direct use of the ABE blends as biofuels would reduce the high energy requirements and the costs related to the separation process of ABE and its components (acetone, butanol, and ethanol), improving the competitiveness of butanol. Considering the low cost of ABE production compared to other biofuels and its potential as a renewable solvent for oils these new biofuels composed of ABE and non-edible vegetable oil represent a promising alternative transportation fuel.

Based on the hopeful results here obtained and since there is not any study about these kinds of fuel blends, it would be interesting to deepen their investigation in the future.

## Figures and Tables

**Table 1:** Technical specifications of the diesel engine electric generator set.

Model	AYERBE 4000 Diesel
Alternator	LINZ-SP 10MF 4.2 KVA
Engine	YANMAR LN-70
Type	Vertical cylinder, 4-cycle, air-cooled diesel engine
Combustion	Direct injection
Bore × Stroke	78 × 67 mm
Displacement	320 cm^3^
Horsepower	6.7
Rated power output	4.5 kW/3000 rpm
Voltage	230 V
Fuel consumption	1.3 L/H (75%)

**Table 2:** Accuracy of the measurements for different parameters.

Parameter	Measuring Range	Accuracy
Soot	0.0–2.5 (Bosch Number); 0 to 50 mg/m^3^	±0.5 mg/m^3^
CO	0–10,000 ppm	±10 ppm (0–200 ppm); ±20 ppm (201–10,000 ppm)
NO	0–300 ppm	±2 ppm
NO_2_	0–500 ppm	±10 ppm
O_2_	0–25 Vol.%	±0.2 Vol.%

**Table 3:** Physicochemical properties of the different components of the fuel blends [23,27,35]. The kinematic viscosity values were experimentally determined in the present research.

Property	Diesel	Sunflower Oil	Castor Oil	Acetone	Butanol	Ethanol
Density at 15 °C (kg/m^3^)	820	920	962	791	811.5	792
Kinematic viscosity at 40 °C (cSt)	3.20 ± 0.03 ^1^	37.80 ± 0.46	226.20 ± 0.55	0.34 ± 0.01	2.27 ± 0.02	1.13 ± 0.01
Calorific value (MJ/L)	35.1	34.3	35.5	23.41	26.94	21.23
Flash point (°C)	66	220	228	−20	35	13
Auto-ignition temperature (°C)	250	316	448	465	397	423
Cetane number	50	37	40	−1	15.92	8

^1^ Errors are expressed as standard deviation from the average of three measurements.

**Table 4:** Viscosity values at 40 °C (cSt, centistokes) for ABE/sunflower oil (SO) and ABE/castor oil (CO) double blends, obtained from the addition of increasing volumes of the ABE mixture to sunflower oil or castor oil. Errors expressed as standard deviation have been calculated from the average of three measurements.

Property	Blend	ABE (% by Volume)						
		0	10	30	50	60	70	100
Kinematic viscosity (cSt)	ABE/SO	37.80 ± 0.46	17.37 ± 0.26	8.65 ± 0.06	4.30 ± 0.10	3.69 ± 0.03	2.27 ± 0.06	1.20 ± 0.01
	ABE/CO	226.20 ± 0.55	102.60 ± 0.31	32.44 ± 0.09	12.41 ± 0.12	8.71 ± 0.05	3.42 ± 0.10	1.20 ± 0.02

**Table 5:** Viscosity values at 40 °C (cSt, centistokes), density, cloud point, pour point, calorific value, and cetane number of the diesel/ABE/sunflower oil blends, obtained by adding different amounts of ABE/sunflower oil double blend, containing 50% ABE, to diesel. Errors are always calculated as standard deviation from the average of three measurements.

Fuel Blend	D/ABE/SO	Density (kg/m^3^)	Kinematic Viscosity (cSt)	Cloud point (°C)	Pour Point (°C)	Calorific Value (MJ/L) ^1^	Cetane Number ^1^
B0	100/0/0	820 ± 6	3.20 ± 0.04	−6.0 ± 1.0	−16.0 ± 1.2	35.10	51.00
B20	80/10/10	832 ± 3	3.22 ± 0.04	−8.3 ± 0.8	−19.0 ± 2.7	34.04	45.51
B40	60/20/20	837 ± 6	3.25 ± 0.04	−9.0 ± 1.6	−20.0 ± 3.0	32.98	40.01
B60	40/30/30	852 ± 4	3.34 ± 0.04	−10.0 ± 1.0	−22.5 ± 2.5	31.92	34.52
B80	20/40/40	857 ± 6	3.75 ± 0.07	−10.6 ± 1.1	−23.5 ± 3.2	30.86	29.02
B100	0/50/50	865 ± 1	4.30 ± 0.10	−13.0 ± 0.7	−24.0 ± 2.3	29.81	23.53

^1^ Calorific value and cetane number were calculated by using Equations (2) and (3), respectively.

**Table 6:** Viscosity values at 40 °C (cSt, centistokes), density, cloud point, pour point, calorific value, and cetane number of the diesel/ABE/castor oil blends, obtained by adding different amounts of ABE/castor oil double blend, containing 70% ABE, to diesel. Errors are always calculated as standard deviation from the average of three measurements.

Fuel Blend	D/ABE/CO	Density (Kg/m^3^)	Kinematic Viscosity (cSt)	Cloud Point (°C)	Pour Point (°C)	Calorific Value (MJ/L) ^1^	Cetane Number ^1^
B0	100/0/0	820 ± 6	3.20 ± 0.04	−6.0 ± 1.0	−16.0± 1.0	35.10	51.00
B20	80/14/6	831 ± 6	3.21 ± 0.04	−11.3 ± 2.8	−23.0 ± 2.0	33.75	44.61
B40	60/28/12	834 ± 8	3.27 ± 0.04	−12.0 ± 0.4	−24.0 ± 2.0	32.41	38.21
B60	40/42/18	839 ± 4	3.31 ± 0.09	−15.0 ± 0.8	−26.5 ± 1.5	31.06	31.82
B80	20/56/24	841 ± 8	3.36 ± 0.02	−16.0 ± 0.7	−28.0 ± 0.7	29.71	25.43
B100	0/70/30	843 ± 6	3.42 ± 0.04	−18.0 ± 1.5	−30.0 ± 0.5	28.37	19.04

^1^ Calorific value and cetane number were calculated by using Equations (2) and (3), respectively.

**Table 7:** Comparison of exhaust emissions generated by a diesel engine fueled with different blends reported in the literature and the percentage of replaced diesel achieved by each of them.

Nomenclature	Blend	Diesel Replacement (%)	Emissions *	Reference
B40SO	60% Diesel/20% ABE/20% CO	40	↓ Soot 69% ↓ NOx 30% ↑ CO 35% ↓ CO_2_ 28% ↓ (slight) power output ≅ BSFC	This study
ABE20	80% Diesel/20% ABE	20	↓ Soot 47% ↑ NOx ↓(slight) power output ↑ BTE	[53]
ABE20	80% Diesel/20% ABE	20	↓ Soot ↑ NOx ↑ BTE	[65]
20ABE80D	80% Diesel/20% ABE	20	↓ Soot 20% ↑ NOx ↓ CO 32–57% ≅ CO_2_ ≅ power output ↑ BSFC	[51,52]
B25A25	50% diesel/25% biodiesel/25% water-containing ABE solution	50	↓ NOx 30.7% ↓ PM 63.1% ↓ PAH 67.6% ↑ BSFC ↑ BTE 7.9%	[55]

* Comparison with diesel. ≅ Comparable ↑ Increased ↓ decreased.
